# BAP1 haploinsufficiency predicts a distinct immunogenic class of malignant peritoneal mesothelioma

**DOI:** 10.1186/s13073-019-0620-3

**Published:** 2019-02-18

**Authors:** Raunak Shrestha, Noushin Nabavi, Yen-Yi Lin, Fan Mo, Shawn Anderson, Stanislav Volik, Hans H. Adomat, Dong Lin, Hui Xue, Xin Dong, Robert Shukin, Robert H. Bell, Brian McConeghy, Anne Haegert, Sonal Brahmbhatt, Estelle Li, Htoo Zarni Oo, Antonio Hurtado-Coll, Ladan Fazli, Joshua Zhou, Yarrow McConnell, Andrea McCart, Andrew Lowy, Gregg B. Morin, Tianhui Chen, Mads Daugaard, S. Cenk Sahinalp, Faraz Hach, Stephane Le Bihan, Martin E. Gleave, Yuzhuo Wang, Andrew Churg, Colin C. Collins

**Affiliations:** 1 Vancouver Prostate Centre, 2660 Oak St, Vancouver, BC V6H 3Z6 Canada; 2 Bioinformatics Training Program, University of British Columbia, Vancouver, BC V6T 1Z4 Canada; 3 Department of Urologic Sciences, University of British Columbia, Vancouver, BC V5Z 1M9 Canada; 4 Department of Surgery, University of British Columbia, Vancouver, BC V5Z 1M9 Canada; 5 BC Cancer Research Centre, 675 W 10th Ave, Vancouver, BC V5Z 1L3 Canada; 6 International Precision Medicine Research Centre, Zhejiang-California International Nanosystems Institute, Zhejiang University, Hangzhou, 310058 Zhejiang China; 7 Neoantigen Therapeutics, Inc., Hangzhou, 310051 Zhejiang China; 8 Mount Sinai Hospital, 600 University Ave, Toronto, ON M5G 1X5 Canada; 9 Moores Cancer Center, 3855 Health Sciences Dr, La Jolla, CA 92093 USA; 10 Zhejiang Academy of Medical Sciences, Tianmushan Road 182, Hangzhou, 310013 China; 11 School of Informatics and Computing, Indiana University, Bloomington, IN 47408 USA; 12 Department of Pathology, Vancouver General Hospital, Vancouver, BC V5Z 1M9 Canada

**Keywords:** Peritoneal mesothelioma, BAP1, Genomics, Tumor immunosurveillance, Precision oncology

## Abstract

**Background:**

Malignant peritoneal mesothelioma (PeM) is a rare and fatal cancer that originates from the peritoneal lining of the abdomen. Standard treatment of PeM is limited to cytoreductive surgery and/or chemotherapy, and no effective targeted therapies for PeM exist. Some immune checkpoint inhibitor studies of mesothelioma have found positivity to be associated with a worse prognosis.

**Methods:**

To search for novel therapeutic targets for PeM, we performed a comprehensive integrative multi-omics analysis of the genome, transcriptome, and proteome of 19 treatment-naïve PeM, and in particular, we examined *BAP1* mutation and copy number status and its relationship to immune checkpoint inhibitor activation.

**Results:**

We found that PeM could be divided into tumors with an inflammatory tumor microenvironment and those without and that this distinction correlated with haploinsufficiency of *BAP1*. To further investigate the role of *BAP1*, we used our recently developed cancer driver gene prioritization algorithm, HIT’nDRIVE, and observed that PeM with *BAP1* haploinsufficiency form a distinct molecular subtype characterized by distinct gene expression patterns of chromatin remodeling, DNA repair pathways, and immune checkpoint receptor activation. We demonstrate that this subtype is correlated with an inflammatory tumor microenvironment and thus is a candidate for immune checkpoint blockade therapies.

**Conclusions:**

Our findings reveal *BAP1* to be a potential, easily trackable prognostic and predictive biomarker for PeM immunotherapy that refines PeM disease classification. *BAP1* stratification may improve drug response rates in ongoing phases I and II clinical trials exploring the use of immune checkpoint blockade therapies in PeM in which *BAP1* status is not considered. This integrated molecular characterization provides a comprehensive foundation for improved management of a subset of PeM patients.

**Electronic supplementary material:**

The online version of this article (10.1186/s13073-019-0620-3) contains supplementary material, which is available to authorized users.

## Background

Malignant mesothelioma is a rare but aggressive cancer that arises from the internal membrane lining of the pleura and the peritoneum. While the majority of mesotheliomas are pleural in origin, the incidence of peritoneal mesothelioma (PeM) accounts for approximately 20–30% of all mesothelioma cases in the USA and possibly higher in other countries [1]. Occupational asbestos exposure is a significant risk factor in the development of pleural mesothelioma (PM). However, epidemiological studies suggest that unlike PM, asbestos exposure plays a far smaller role in the etiology of PeM tumors [2]. More importantly, the incidence of PeM is skewed towards young women of childbearing ages rather than in old patients [1] making PeM a malignancy often associated with many years of life lost.

Previous studies in mesotheliomas have revealed that over 60% of mesotheliomas harbor *BRCA1* associated protein 1 (*BAP1*) inactivating mutation or copy number loss, making *BAP1* the most commonly altered gene in this malignancy [3–7]. BAP1 is a tumor suppressor and deubiquitinase, localized to the nucleus, known to regulate chromatin remodeling and maintain genome integrity [8, 9]. Furthermore, BAP1 localized in endoplasmic reticulum regulate calcium (Ca^2+^) flux to promote apoptosis [10]. Thus, the combined reduced BAP1 nuclear and cytoplasmic activity results in the accumulation of DNA-damaged cells and greater susceptibility to the development of malignancy. In addition, inactivating mutations of neurofibromin 2 (*NF2*) and cyclin-dependent kinase inhibitor 2A (*CDKN2A*) are also relatively common, while other mutations are rare. Previous studies in PeM [11–18] have only focused on genomic information; therefore, the downstream consequences of these genomic alterations are not well understood. Genome information coupled with transcriptome and proteome information is more likely to be successful in revealing potential therapeutic modalities.

Mesothelioma is typically diagnosed in the advanced stages of the disease. A combination of cytoreductive surgery (CRS) and hyperthermic intraperitoneal chemotherapy (HIPEC), sometimes followed by normothermic intraperitoneal or systemic chemotherapy (NIPEC), has recently emerged as the first-line treatment for PeM [19]. However, even with this regime, complete cytoreduction is hard to achieve and death ensues for many patients. Actionable molecular targets for PeM critical for precision oncology remain to be defined. Immune checkpoint blockade therapy in PM has recently gained traction [7, 20] given that 20–40% of PM cases are reported to show an inflammatory phenotype [21]. However, the role of immunostaining for PD-L1, the usual approach to predicting a response to immunotherapy for other tumor types, is controversial in PM, since positive stating has generally been associated with a worse prognosis, and it is unclear what marker should be used to predict tumors that may respond to immunotherapy.

Although, clinical trials typically lump PeM and PM together for immune checkpoint blockade [22–26], even less is known about PeM and immunotherapy. Thus, there has been no attempt to stratify PeM patients. In this study, we performed an integrated multi-omics analysis of the genome, transcriptome, and proteome of 19 PeM, predominantly of epithelioid subtype, and correlated these with tumor inflammation.

## Methods

### Patient cohort

We assembled a cohort of 19 PeM from 18 patients (Table 1 and Additional file 2: Table S1) undergoing CRS at Vancouver General Hospital (Vancouver, Canada), Mount Sinai Hospital (Toronto, Canada), and Moores Cancer Centre (San Diego, CA, USA). We obtained 19 fresh-frozen primary treatment-naïve PeM tumor tissue and adjacent benign tissues or whole blood from the 18 patients. For 1 patient, MESO-18, 2 tumors from distinct sites were available. Immunohistochemical analyses using different biomarkers were evaluated by 2 independent pathologists (Additional file 1: Figure S1-S4). Both pathologists categorized all 19 tumors as epithelioid PeM with a content of higher than 75% tumor cellularity. To the best of our knowledge, this is the largest cohort of PeM subjected to an integrative multi-omics analysis.

**Table 1:** Peritoneal mesothelioma patients recruited for the study

Tumor	Asbestos exposure	Subtype	WES	WTS	MS
MESO-01	Unknown	BAP1-intact	Yes	No	Yes
MESO-02	Unknown	BAP1-del	Yes	Yes	Yes
MESO-03	Unknown	BAP1-intact	Yes	No	Yes
MESO-04	Unknown	BAP1-intact	Yes	No	Yes
MESO-05	Unknown	BAP1-del	Yes	Yes	Yes
MESO-06	No	BAP1-del	Yes	Yes	Yes
MESO-07	Unknown	BAP1-del	Yes	Yes	Yes
MESO-08	No	BAP1-intact	Yes	Yes	No
MESO-09	No	BAP1-del	Yes	Yes	Yes
MESO-10	No	BAP1-del	Yes	Yes	Yes
MESO-11	No	BAP1-intact	Yes	Yes	Yes
MESO-12	No	BAP1-intact	Yes	Yes	Yes
MESO-13	No	BAP1-intact	Yes	Yes	Yes
MESO-14	No	BAP1-del	Yes	Yes	Yes
MESO-15	No	BAP1-intact	Yes	No	No
MESO-17	No	BAP1-del	Yes	Yes	Yes
MESO-18A	No	BAP1-intact	Yes	Yes	Yes
MESO-18E	No	BAP1-intact	Yes	Yes	Yes
MESO-19	Yes	BAP1-intact	Yes	Yes	No

*WES* whole exome sequencing, *WTS* whole transcriptome sequencing, *MS* mass spectrometry

### Immunohistochemistry and histopathology

Freshly cut tissue microarray (TMA) sections were analyzed for immunoexpression using Ventana Discovery Ultra autostainer (Ventana Medical Systems, Tucson, AZ). In brief, tissue sections were incubated in Tris-EDTA buffer (CC1) at 37 °C to retrieve antigenicity, followed by incubation with respective primary antibodies at room temperature or 37 °C for 60–120 min. For primary antibodies, mouse monoclonal antibodies against CD8 (Leica, NCL-L-CD8-4B11, 1:100), CK5/cytokeratin 5 (Abcam, ab17130, 1:100), BAP1 (SantaCruz, clone C4, sc-28383, 1:50), rabbit monoclonal antibody against CD3 (Abcam, ab16669, 1:100), and rabbit polyclonal antibodies against CALB2/calretinin (LifeSpan BioSciences, LS-B4220, 1:20 dilution) were used. Bound primary antibodies were incubated with Ventana Ultra HRP kit or Ventana universal secondary antibody and visualized using Ventana ChromoMap or DAB Map detection kit, respectively. All stained slides were digitalized with the SL801 autoloader and Leica SCN400 scanning system (Leica Microsystems; Concord, Ontario, Canada) at magnification equivalent to × 20. The images were subsequently stored in the SlidePath digital imaging hub (DIH; Leica Microsystems) of the Vancouver Prostate Centre. Representative tissue cores were manually identified by two pathologists.

### Whole exome sequencing

DNA was isolated from snap-frozen tumors with 0.2 mg/ml Proteinase K (Roche) in a cell lysis solution using Wizard Genomic DNA Purification Kit (Promega Corporation, USA). Digestion was carried out overnight at 55 °C before incubation with RNase solution at 37 °C for 30 min and treatment with protein precipitation solution followed by isopropanol precipitation of the DNA. The amount of DNA was quantified on the NanoDrop 1000 Spectrophotometer and an additional quality check done by reviewing the 260/280 ratios. Quality check was done on the extracted DNA by running the samples on a 0.8% agarose/TBE gel with ethidium bromide.

For Ion AmpliSeq™ Exome Sequencing, 100 ng of DNA based on Qubit® dsDNA HS Assay (Thermo Fisher Scientific) quantitation was used as input for Ion AmpliSeq™ Exome RDY Library preparation. This is a polymerase chain reaction (PCR)-based sequencing approach using 294,000 primer pairs (amplicon size range 225–275 bp) and covers > 97% of Consensus CDS (CCDS; release 12), > 19,000 coding genes, and > 198,000 coding exons. Libraries were prepared, quantified by quantitative PCR (qPCR), and sequenced according to the manufacturer’s instructions (Thermo Fisher Scientific). Samples were sequenced on the Ion Proton System using the Ion PI™ Hi-Q™ Sequencing 200 Kit and Ion PI™ v3 chip. Two libraries were run per chip for a projected coverage of 40 M reads per sample.

### Somatic variant calling

Torrent Server (Thermo Fisher Scientific) was used for signal processing, base calling, read alignment, and generation of results files. Specifically, following sequencing, reads were mapped against the human reference genome hg19 using the Torrent Mapping Alignment Program. Variants were identified by using Torrent Variant Caller plugin with the optimized parameters for AmpliSeq exome-sequencing recommended by Thermo Fisher. The variant call format (VCF) files from all samples were combined using GATK (3.2-2) [27], and all variants were annotated using ANNOVAR [28]. Only non-silent exonic variants including non-synonymous single nucleotide variations (SNVs), stop-codon gain SNVs, stop-codon loss SNVs, splice site SNVs, and In-Dels in coding regions were kept if they were supported by more than ten reads and had allele frequency higher than 10%. To obtain somatic variants, we filtered against dbSNP build 138 (non-flagged only) and the matched adjacent benign or blood samples sequenced in this study. Putative variants were manually scrutinized on the Binary Alignment Map (BAM) files through Integrative Genomics Viewer version 2.3.25 [29].

### Copy number aberration (CNA) analysis

Copy number changes were assessed using Nexus Copy Number Discovery Edition version 9.0 (BioDiscovery, Inc., El Segundo, CA). Nexus NGS functionality (BAM ngCGH) with the FASST2 segmentation algorithm was used to make copy number calls (a circular binary segmentation/hidden Markov model approach). The significance threshold for segmentation was set at 5 × 10^−6^, also requiring a minimum of 3 probes per segment and a maximum probe spacing of 1000 between adjacent probes before breaking a segment. The log ratio thresholds for single copy gain and single copy loss were set at + 0.2 and − 0.2, respectively. The log ratio thresholds for the gain of 2 or more copies and for a homozygous loss were set at + 0.6 and − 1.0, respectively. Tumor sample BAM files were processed with corresponding normal tissue BAM files. Reference reads per CNA point (window size) was set at 8000. Probes were normalized to the median. Relative copy number profiles from exome sequencing data were determined by normalizing tumor exome coverage to values from whole blood controls.

### Whole transcriptome sequencing (RNA-seq)

Total RNA from 100 μm sections of snap-frozen tissue was isolated using the mirVana Isolation Kit from Ambion (AM-1560). Strand-specific RNA sequencing was performed on quality controlled high RIN value (> 7) RNA samples (Bioanalyzer Agilent Technologies) before processing at the high throughput sequencing facility core at BGI Genomics Co., Ltd. (The Children’s Hospital of Philadelphia, PA, USA). In brief, 200 ng of total DNAse-treated RNA was first treated to remove the ribosomal RNA (rRNA) and then purified using the Agencourt RNA Clean XP Kit (Beckman Coulter) prior to analysis with the Agilent RNA 6000 Pico Chip to confirm rRNA removal. Next, the rRNA-depleted RNA was fragmented and converted to cDNA. Subsequent steps include end repair, addition of an “A” overhang at the 3′ end, and ligation of the indexing-specific adaptor, followed by purification with Agencourt Ampure XP beads. The strand-specific RNA library prepared using TruSeq (Illumina catalog no. RS-122-2201) was amplified and purified with Ampure XP beads. Size and yield of the barcoded libraries were assessed on the LabChip GX (Caliper), with an expected distribution around 260 bp. The concentration of each library was measured with real-time PCR. Pools of the indexed library were then prepared for cluster generation and PE100 sequencing on Illumina HiSeq 4000. The RNA-seq reads were aligned using STAR (2.3.1z) [30] onto the human genome reference (GRCh38), and the transcripts were annotated based on Ensembl release 80 gene models. Only the reads unique to one gene and which corresponded exactly to one gene structure were assigned to the corresponding genes by using HTSeq [31]. Normalization of the read counts was conducted by DESeq [32]. For a detailed description, see Additional file 1: Supplementary Methods.

### Proteomics analysis using mass spectrometry

Fresh-frozen samples dissected from tumor and adjacent normal were individually lysed in 50 mM of HEPES pH 8.5, 1% SDS, and the chromatin content was degraded with Benzonase. The tumor lysates were sonicated (Bioruptor Pico, Diagenode, NJ, USA), and disulfide bonds were reduced with DTT and capped with iodoacetamide. Proteins were cleaned up using the SP3 method [33, 34] (Single Pot, Solid Phase, Sample Prep) then digested overnight with trypsin in HEPES pH 8, peptide concentration determined by Nanodrop (Thermo) and adjusted to an equal level. A pooled internal standard control was generated comprising of equal volumes of every sample (10 μl of each of the 100 μl total digests) and split into 3 equal aliquots. The labeling reactions were run as 3 TMT 10-plex panels (9+IS) then desalted, and each panel is divided into 48 fractions by reverse-phase HPLC at pH 10 with an Agilent 1100 LC system. The 48 fractions were concatenated into 12 superfractions per panel by pooling every fourth fraction eluted resulting in a total of 36 overall samples. These samples were analyzed with an Orbitrap Fusion Tribrid Mass Spectrometer (Thermo Fisher Scientific) coupled with EasyNanoLC 1000 using a data-dependent method with synchronous precursor selection (SPS) MS3 scanning for TMT tags. Based on ProteomeDiscoverer 2.1.1.21 (Thermo Fisher Scientific), we selected peptide-spectrum match (PSM) results with *q* value ≤ 0.05 and extract proteins from both high and medium confidence level after false discovery rate filtering for protein identification and quantification results. For a detailed description, see Additional file 1: Supplementary Methods.

### Prioritization of driver genes using HIT’nDRIVE

Non-silent somatic mutation calls, CNA gain or loss, and gene-fusion calls were collapsed in gene-patient alteration matrix with binary labels. Gene expression values were used to derive an expression-outlier gene-patient outlier matrix using the generalized extreme studentized deviate (GESD) test. STRING ver10 [35] protein interaction network was used to compute pairwise influence value between the nodes in the interaction network. We integrated these genome and transcriptome data using the HIT’nDRIVE algorithm [36]. The following parameters were used: *α* = 0.9, *β* = 0.6, and *γ* = 0.8. We used IBM-CPLEX as the integer linear programming (ILP) solver.

### Stromal and immune score

We used 2 sets of 141 genes (1 each for stromal and immune gene signatures) as described in [37]. We used the “inverse normal transformation” method to transform the distribution of expression data into the standard normal distribution. The stromal and immune scores were calculated, for each sample, using the summation of standard normal deviates of each gene in the given set.

### Enumeration of tissue-resident immune cell types using mRNA expression profiles

CIBERSORT software [38] was applied to the RNA-seq gene expression data to estimate the proportions of 22 immune cell types (B cells naive, B cells memory, plasma cells, T cells CD8, T cells CD4 naive, T cells CD4 memory resting, T cells CD4 memory activated, T cells follicular helper, T cells gamma delta, T cells regulatory (Tregs), NK cells resting, NK cells activated, monocytes, macrophages M0, macrophages M1, macrophages M2, dendritic cells resting, dendritic cells activated, mast cells resting, mast cells activated, eosinophils, and neutrophils) using LM22 dataset provided by CIBERSORT platform. Genes not expressed in any of the PeM tumor samples were removed from the LM22 dataset. The analysis was performed using 1000 permutations. The 22 immune cell types were later aggregated into 9 distinct groups.

## Results

### Landscape of somatic mutations in PeM

To investigate the landscape of somatic gene mutations in PeM, we performed high-coverage whole exome sequencing of 19 tumors and 16 matched normal samples (Additional file 2: Table S1). We achieved a mean coverage of 180× for cancerous samples and 96× for non-cancerous samples (Additional file 2: Table S2). We identified 346 unique non-silent mutations affecting 202 unique genes (Additional file 1: Figure S5 and Additional file 2: Table S3). We observed an average of 0.015 protein-coding non-silent mutations per Mb per tumor sample.

**Fig. 1:**
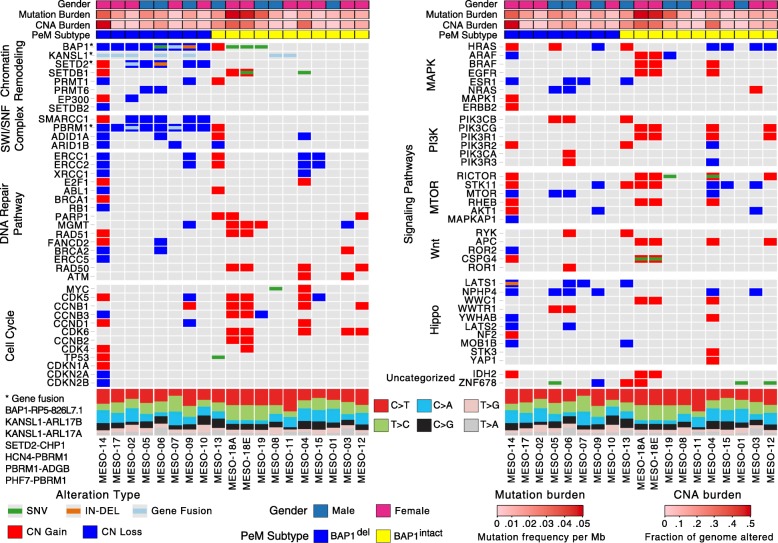
Integrated molecular comparison of somatic alterations across peritoneal mesothelioma subtypes. Somatic alterations status in PeM subtypes grouped by important cancer-pathways—chromatin remodeling, SWI/SNF complex, DNA repair pathway, cell cycle, MAPK, PI3K, MTOR, Wnt, and Hippo pathways. Somatic mutation status, copy number status, gene fusion, distribution of substitution mutation types, mutation burden, and copy number aberration burden are indicated

We first identified driver genes of PeM using our recently developed computational algorithm HIT’nDRIVE [36]. Briefly, HIT’nDRIVE measures the potential impact of genomic aberrations on changes in the global expression of other genes/proteins which are in close proximity in a gene/protein interaction network. It then prioritizes those aberrations with the highest impact as cancer driver genes. HIT’nDRIVE prioritized 25 unique driver genes in 15 PeM samples for which matched genome and transcriptome data were available (Fig. 1 and Additional file 2: Table S4). Six genes (*BAP1*, *BZW2*, *ABCA7*, *TP53*, *ARID2*, and *FMN2*) were prioritized as drivers, harboring single nucleotide changes.

**Fig. 2:**
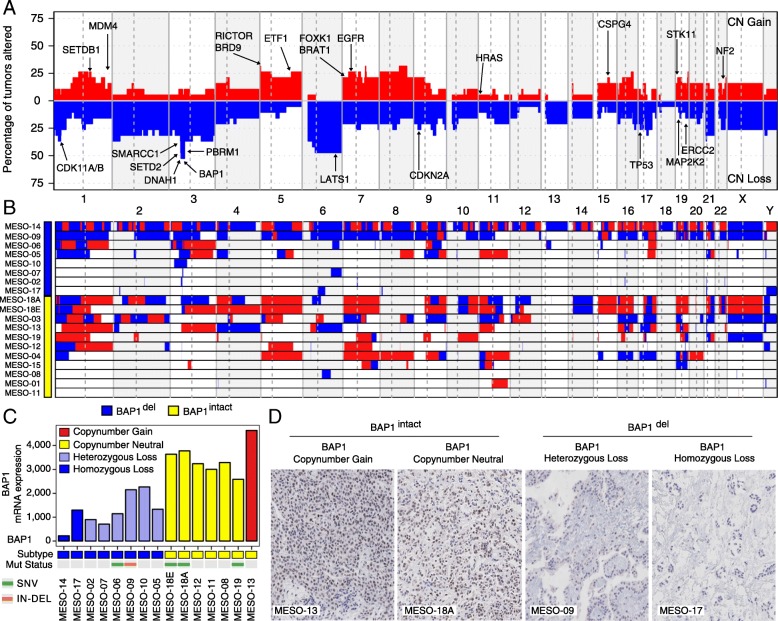
Landscape of copy number aberrations in PeM. **a** Aggregate copy number alterations by chromosome regions in PeM. Important genes with copy number changes are highlighted. **b** Sample-wise view of copy number alterations in PeM. **c** mRNA expression pattern of *BAP1* across all PeM samples. **d** Detection of *BAP1* nuclear protein expression in PeM tumors by immunohistochemistry (photomicrographs magnification, × 20)

*BAP1* was the most frequently mutated gene (5 out of 19 tumors) in PeM. Among the 5 *BAP1*-mutated cases, 2 cases (MESO-06 and MESO-09) were predicted to have inactivated BAP1, whereas despite *BAP1* mutation in 3 cases (MESO-18A/E and MESO-19), their mRNA transcripts were expressed in high levels (Fig. 2c and Additional file 1: Figure S6-S7). We identified that all variants of *BAP1* (except a 42-bp deletion in MESO-09) were expressed at the RNA level (Additional file 2: Table S16). In addition, we identified mutations in genes such as *TP53*, *SETD2*, *SETDB1*, and *LATS1* each present in just a single case (Fig. 1).

### Copy number landscape in PeM

The aggregate somatic copy number aberration (CNA) profile of PeM is shown in Fig. 2a, b. We observed a total of 1281 CNA events across all samples (Additional file 2: Table S5). On average, 10% of the protein-coding genome was altered per PeM. Interestingly, the CNA burden in PeM was strongly correlated (*R* = 0.74) with its mutation burden (Additional file 1: Figure S9).

Using HIT’nDRIVE, we identified genes in chromosomes 3p21, *BAP1*, *PBRM1*, and *SETD2*, as key driver genes of PeM (Fig. 1 and Additional file 2: Table S4). This region was also identified as significantly recurrent focal CNAs using the GISTIC [39] algorithm (Additional file 1: Figure S9). Chromosome 3p21 was deleted (homozygous or heterozygous) in almost half of the tumors (8 of 19) in the cohort. Here, we call tumors with 3p21 (or *BAP1*) loss as *BAP1*^del^ and the rest of the tumors with 3p21 (or *BAP1*) copy number intact as *BAP1*^intact^. Interestingly, *BAP1* mRNA transcripts in *BAP1*^del^ tumors were expressed at lower levels as compared to those in *BAP1*^intact^ tumors (*p* value = 3 × 10^−4^) (Fig. 2c). We validated this using immunohistochemical (IHC) staining demonstrating a lack of BAP1 nuclear staining in the tumors with *BAP1* homozygous deletion (Fig. 2d). Tumors with *BAP1* heterozygous loss still displayed BAP1 nuclear staining (Additional file 1: Figure S10). We observed 3 *BAP1*-mutated cases (MESO-18A/E and MESO-19) among *BAP1*^intact^ tumors. *BAP1* mRNA transcripts in these 3 tumors were expressed at high levels (Fig. 2c). Furthermore, we found DNA copy loss of 3p21 locus to include 4 concomitantly deleted cancer genes—*BAP1*, *SETD2*, *SMARCC1*, and *PBRM1*—consistent with [5]. Copy number status of these 4 genes was significantly correlated with their corresponding mRNA expression (Additional file 1: Figure S11), suggesting that the allelic loss of these genes is associated with decreased transcript levels. These 4 genes are chromatin modifiers, and *PBRM1* and *SMARCC1* are part of the SWI/SNF complex that regulates transcription of a number of genes.

### The global transcriptome and proteome profile of PeM

To segregate transcriptional subtypes of PeM, we performed total RNA-seq (Illumina HiSeq 4000) and its quantification of 15 PeM tumor samples for which RNA were available. Using principal component analyses, we found that tumor samples in *BAP1*^intact^ and *BAP1*^del^ subtypes have distinct transcriptomic patterns; however, a few samples showed an overlapping pattern (Additional file 1: Figure S16A).

We performed mass spectrometry (Fusion Orbitrap LC/MS/MS) with isobaric tagging for expressed peptide identification and its corresponding protein quantification using Proteome Discoverer for processing pipeline for 16 PeM tumors and 7 matched normal tissues. We identified 8242 unique proteins in 23 samples analyzed. We were surprised BAP1 protein was however not detected in our MS experiment, likely due to inherent technical limitations with these samples and/or processing. Quality control analysis of in-solution Hela digests also has very low BAP1 with only a single peptide observed in occasional runs. Unlike in transcriptome profiles, the proteome profiles of tumor samples in *BAP1*^intact^ and *BAP1*^del^ subtypes did not group into distinct clusters (Additional file 1: Figure S16B).

Next, we identified differentially expressed genes and proteins between *BAP1*^intact^ and *BAP1*^del^ subtypes (see Additional file 1: Supplemental Methods). As expected, *BAP1*, *PBRM1*, *SMARCA4*, and *SMARCD3* were among the top 500 differentially expressed genes. Many other important cancer-related genes were differentially expressed such as *CDK20*, *HIST1H4F*, *ERCC1*, *APOBEC3A*, *CDK11A*, *CSPG4*, *TGFB1*, *IL6*, *LAG3*, and *ATM*.

**Fig. 3:**
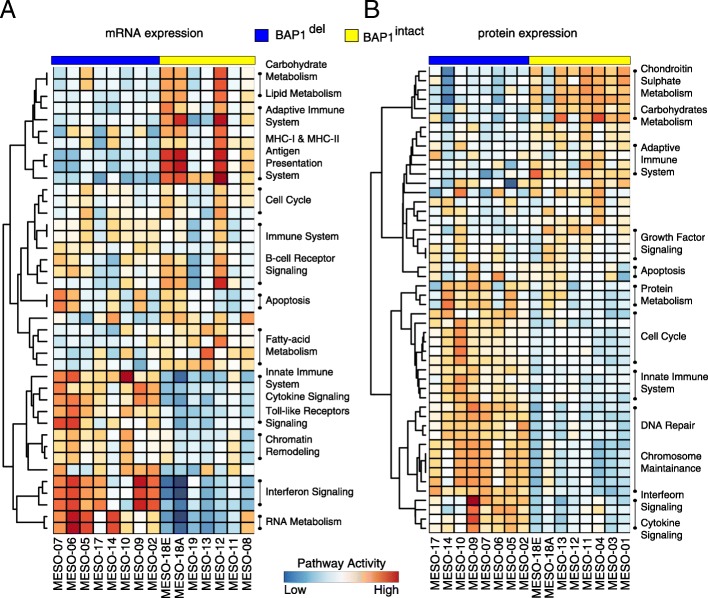
Transcriptome and proteome profile of PeM. Pathway enrichment of top 500 differentially expressed genes between PeM subtypes obtained using **a** mRNA expression and **b** protein expression. The colors on the heatmap show the pathway activity of the respective signaling pathways

To identify the pathways dysregulated by the differentially expressed genes between the PeM subtypes, we performed gene set enrichment analysis (see Additional file 1: Supplementary Methods). Intriguingly, we observed high concordance between pathways dysregulated by the 2 sets (mRNA and protein expression data) of top 500 differentially expressed genes and proteins (Fig. 3a, b). The unsupervised clustering of pathways revealed 2 distinct clusters for *BAP1*^del^ and *BAP1*^intact^ tumors. This indicates that the enriched pathways, between the patient groups, are also differentially expressed. *BAP1*^del^ tumors demonstrated elevated levels of RNA and protein metabolism as compared to *BAP1*^intact^ tumors. Many genes involved in chromatin remodeling and DNA damage repair were differently expressed between the groups (Additional file 1: Figure S20-S21). Genes in DNA damage repair pathway—*PARP1*, *ERCC1*, and *APC*—were downregulated, and *CHEK1/2*, *BRAC2*, *RAD50*, and *ATM* were upregulated in *BAP1*^del^ tumors. Genes involved in cell cycle and apoptotic pathways were observed to be highly expressed in *BAP1*^del^ patients. Furthermore, glucose and fatty acid metabolism pathways were repressed in *BAP1*^del^ as compared to *BAP1*^intact^. More interestingly, we observed a striking difference in immune system-associated pathways between the PeM subtypes, whereas *BAP1*^del^ tumors demonstrated strong activity of cytokine signaling and the innate immune system; the MHC-I/II antigen presentation system and adaptive immune system were active in *BAP1*^intact^ tumors.

### *BAP1*^del^ subtype is correlated with tumor inflammation characterized by immune checkpoint receptor activation

**Fig. 4:**
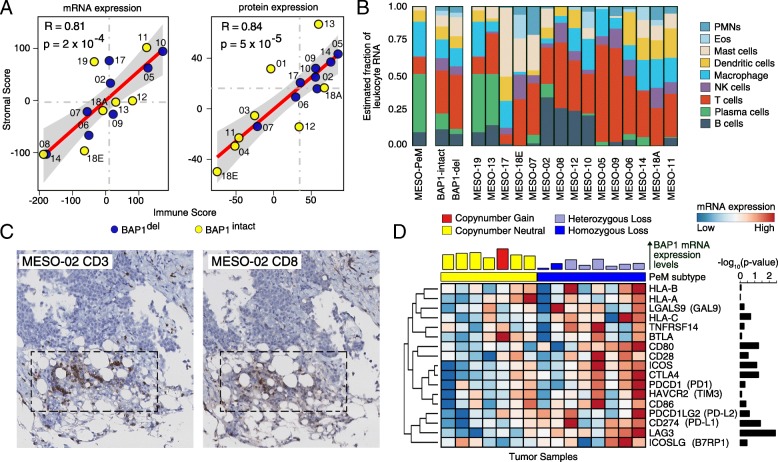
Immune cell infiltration in PeM. **a** Correlation between immune score and stromal score derived for each tumor sample using mRNA expression and protein expression. **b** Estimated relative mRNA fractions of leukocytes infiltrated in PeM tumors based on CIBERSORT analysis. **c** CD3 and CD8 immunohistochemistry showing immune cell infiltration on *BAP1*^del^ PeM (photomicrographs magnification, × 20). **d** mRNA expression differences in immune checkpoint receptors—*LAG3*, *PD1*, *CTLA4*, *CD28*, *ICOS*, *BTLA*, and *HAVCR2* between PeM subtypes. Other genes in the figures are interacting receptors of the immune checkpoint markers mentioned above. The bar plot of the top of the heatmap indicates *BAP1* mRNA expression levels. The colors on the bar indicate *BAP1* copy number status. The bar plot on the right represents the negative log10 of Wilcoxon signed-rank test *p* value of individual immune checkpoint receptors computed between PeM subtypes. The expression levels are log2 transformed and mean normalized

Prompted by this finding, we next analyzed whether PeM were infiltrated with leukocytes. To assess the extent of leukocyte infiltration, we computed an expression-based (RNA-seq and protein) score (see the “Methods” section) using the immune cell and stromal markers proposed by [37]. We discovered that the immune marker gene score was strongly correlated with the stromal marker gene score (Fig. 4a) suggesting possible leukocyte infiltration in PeM from the tumor microenvironment. Furthermore, using CIBERSORT [38] software, we computationally estimated the leukocyte representation in the bulk tumor transcriptome. We observed massive infiltration of T cells in majority of the PeM (Fig. 4b). A subset of PeM had massive infiltration of B cells in addition to T cells. Interestingly, when we group the PeM by their *BAP1* aberration status, there was a marked difference in the proportion of infiltrated plasma cells, natural killer (NK) cells, mast cells, and B cells between the groups. Whereas the proportions of plasma cells, NK cells, and B cells were less in the *BAP1*^del^ tumors, there was more infiltration of mast cells and T cells in *BAP1*^del^ tumors as compared to *BAP1*^intact^ tumors. We performed TMA IHC staining of CD3 and CD8 antibodies on PeM tumors. We observed that *BAP1*^del^ PeM were positively stained for both CD3 and CD8 confirming infiltration of T cells in *BAP1*^del^ PeM (Fig. 4c and Additional file 1: Figure S22-S23). Combined, this strongly indicates that PeM could be divided into tumors with an inflammatory tumor microenvironment and those without and that this distinction correlated with *BAP1* haploinsufficiency.

Finally, we surveyed PeM for the expression of genes involved in immune checkpoint pathways. A number of immune checkpoint receptors were highly expressed in *BAP1*^del^ tumors relative to *BAP1*^intact^ tumors. These included *PDCD1* (*PD1*), *CD274* (*PD-L1*), *CD80*, *CTLA4*, *LAG3*, and *ICOS* (Fig. 4d and Additional file 1: Figure S30) for which inhibitors are either clinically approved or are at varying stages of clinical trials. Notably, the differential gene expression pattern of *LAG3*, *ICOS*, and *CTLA4* between the PeM subtypes suggests potential opportunities for immune checkpoint blockade beyond conventional PD1/PD-L1. Moreover, a number of MHC genes, immuno-inhibitor genes, and immuno-stimulator genes were differentially expressed between *BAP1*^del^ and *BAP1*^intact^ tumors (Additional file 1: Figure S24). Furthermore, we analyzed whether the immune checkpoint receptors were differentially expressed in tumors with and without 3p21 loss in the pleural mesotheliomas (PM) from The Cancer Gene Atlas (TCGA) project [7]. Unlike in PeM, we did not observe a significant difference in immune checkpoint receptor expression between the PM groups (i.e., *BAP1*^del^ and *BAP1*^intact^) (Additional file 1: Figure S25). These findings suggest that *BAP1*^del^ PeM tumors could potentially be targeted with immune checkpoint inhibitors while PM tumors may less likely to respond.

## Discussion

In this study, we present a comprehensive integrative multi-omics analysis of malignant peritoneal mesotheliomas. Even though this is a rare disease, we managed to amass a cohort of 19 tumors. Prior studies of mesotheliomas, performed using a single omic platform, have established *BAP1* inactivation as a key driver event in mesotheliomas. Our novel contribution to PeM is that we provide evidence from integrative multi-omics analyses that *BAP1* haploinsufficiency (*BAP1*^del^) forms a distinct molecular subtype of PeM. This subtype is characterized by distinct expression patterns of genes involved in chromatin remodeling, DNA repair pathway, and immune checkpoint activation. Moreover, *BAP1*^del^ subtype is correlated with inflammatory tumor microenvironment. Our results suggest that *BAP1*^del^ tumors might be prioritized for immune checkpoint blockade therapies. Thus, *BAP1* is likely both prognostic and predictive biomarker for PeM enabling better disease stratification and patient treatment. Further corroborating our findings, BAP1 status has been recently shown to be correlated with perturbed immune signaling in PM [7].

Loss of *BAP1* is known to alter chromatin architecture exposing the DNA to damage and also impairing the DNA repair machinery [9, 40]. The DNA repair defects thus drive genomic instability and dysregulate tumor microenvironment [41]. DNA repair deficiency leads to the increased secretion of cytokines, including interferons that promote tumor-antigen presentation and trigger recruitment of T lymphocytes to destroy tumor cells. As a response, tumor cells evade this immune surveillance by increased expression of immune checkpoint receptors. The results presented here also indicate that PeM are infiltrated with immune cells from the tumor microenvironment. Moreover, the *BAP1*^del^ subtype displays elevated levels of immune checkpoint receptor expression which strongly suggests the use of immune checkpoint inhibitors to treat this subtype of PeM. However, in a small subset of PM tumors in TCGA dataset, the loss of *BAP1* did not elevate the expression of immune checkpoint marker genes. This warrants further investigation on the characteristics of these groups of PM.

The main challenge in mesothelioma treatment is that all current efforts made towards testing new therapy options are limited to using therapies that have been proven successful in other cancer types, without a good knowledge of underlying molecular mechanisms of the disease. As a result of sheer desperation, some patients have been treated even though no targeted therapy for mesothelioma has been proven effective as yet. For example, a number of clinical trials exploring the use of immune checkpoint blockade (anti-PD1/PD-L1 or anti-CTLA4) in PM and/or PeM patients are currently under progress. The results of the first few clinical trials report either a very low response rate or no benefit to the patients [22–24, 26, 42]. Notably, *BAP1* copy number or mutation status was not assessed in these studies. Our study warrants further investigation of immune checkpoint molecules targeting beyond conventional PD1/PD-L1. We hypothesize based on this evidence presented that response rates for immune checkpoint blockade therapies in clinical trials for PeM will improve when patients are segregated by their *BAP1* copy number status.

## Conclusion

Our first-in-field multi-omics analysis of PeM tumors identified *BAP1* haploinsufficiency as a distinct molecular subtype and a candidate for immune checkpoint blockade therapies. This is significant because almost half of PeM cases are now candidates for these therapies. *BAP1* status is not currently taken into account in the ongoing phases I and II clinical trials exploring the use of immune checkpoint blockade therapies in PeM. Moreover, this is the first study to demonstrate evidence of inflammatory tumor microenvironment in PeM. Our findings identify *BAP1* as a tractable prognostic and predictive biomarker for immunotherapy that refines PeM disease stratification and may improve drug response rates.

## Additional files

Additional file 1: Supplementary material. (PDF 11113 kb)

Additional file 2: Supplementary tables. (XLSX 1031 kb)

## Abbreviations

BAM: Binary alignment map
BAP1: BRCA1-associated protein 1
CDKN2A: Cyclin-dependent kinase inhibitor 2A
CNA: Copy number aberration
CRS: Cytoreductive surgery
GESD: Generalized extreme studentized deviate
HIPEC: Hyperthermic intraperitoneal chemotherapy
IHC: Immunohistochemical
ILP: Integer linear programming
NF2: Neurofibromin 2
NIPEC: Normothermic intraperitoneal chemotherapy
PCR: Polymerase chain reaction
PeM: Peritoneal mesothelioma
PM: Pleural mesothelioma
PSM: Peptide-spectrum match
qPCR: Quantitative PCR
SETD2: SET domain containing 2
SNV: Single nucleotide variation
SPS: Synchronous precursor selection
TMA: Tissue microarray
VCF: Variant call format
